# The effect of warm-up, static stretching and dynamic stretching on hamstring flexibility in previously injured subjects

**DOI:** 10.1186/1471-2474-10-37

**Published:** 2009-04-16

**Authors:** Kieran O'Sullivan, Elaine Murray, David Sainsbury

**Affiliations:** 1 Physiotherapy Department, University of Limerick, Limerick, Ireland; 2 Physical Activity, Occupation and Health Research Unit, University of Limerick, Limerick, Ireland

## Abstract

**Background:**

Warm-up and stretching are suggested to increase hamstring flexibility and reduce the risk of injury. This study examined the short-term effects of warm-up, static stretching and dynamic stretching on hamstring flexibility in individuals with previous hamstring injury and uninjured controls.

**Methods:**

A randomised crossover study design, over 2 separate days. Hamstring flexibility was assessed using passive knee extension range of motion (PKE ROM). 18 previously injured individuals and 18 uninjured controls participated. On both days, four measurements of PKE ROM were recorded: (1) at baseline; (2) after warm-up; (3) after stretch (static or dynamic) and (4) after a 15-minute rest. Participants carried out both static and dynamic stretches, but on different days. Data were analysed using Anova.

**Results:**

Across both groups, there was a significant main effect for time (p < 0.001). PKE ROM significantly increased with warm-up (p < 0.001). From warm-up, PKE ROM further increased with static stretching (p = 0.04) but significantly decreased after dynamic stretching (p = 0.013). The increased flexibility after warm-up and static stretching reduced significantly (p < 0.001) after 15 minutes of rest, but remained significantly greater than at baseline (p < 0.001). Between groups, there was no main effect for group (p = 0.462), with no difference in mean PKE ROM values at any individual stage of the protocol (p > 0.05). Using ANCOVA to adjust for the non-significant (p = 0.141) baseline difference between groups, the previously injured group demonstrated a greater response to warm-up and static stretching, however this was not statistically significant (p = 0.05).

**Conclusion:**

Warm-up significantly increased hamstring flexibility. Static stretching also increased hamstring flexibility, whereas dynamic did not, in agreement with previous findings on uninjured controls. The effect of warm-up and static stretching on flexibility was greater in those with reduced flexibility post-injury, but this did not reach statistical significance. Further prospective research is required to validate the hypothesis that increased flexibility improves outcomes.

**Trial Registration:**

ACTRN12608000638336

## Background

Pre-exercise routines are common practice for the majority of individuals participating in sport. Though the optimal pre-exercise routine is debated [1,2], an active aerobic warm-up is commonly used as it has been shown to improve performance measures [3]. Stretching is also usually incorporated pre-exercise as it has been suggested to improve muscle flexibility, prevent muscle injury and enhance physical performance [4-7]. Hamstring strains are one of the most common, recurrent injuries experienced in the sporting world [8] and often result in significant time out of sport and activity [9]. Decreased hamstring flexibility is suggested to be one of the predisposing factors for hamstring strains [10-14] and hamstring stretches are routinely used as part of a pre-exercise routine, usually after an aerobic warm-up. A static stretch is performed by placing muscles at their greatest possible length and holding that position for a period of time [15]. In contrast, dynamic stretching involves moving the limb from its neutral position to end range, where the muscles are at their greatest length and then moving the limb back to its original position. This dynamic action is carried out in a smooth, controlled manner and is repeated for a specified time period [16].

Previous research suggests static stretching may help reduce injury rates [10,12] and improve recovery from injury [17-19]. However other studies suggest that static stretching has little or no impact on injury prevention [20-22]. It has also become clear that static stretching may negatively affect immediate physical performance [2,23]. Because of this, dynamic stretching has been recommended as an alternative to static stretching post warm-up, as evidence suggests that dynamic stretching positively impacts on immediate physical performance [4,24]. Dynamic stretches, however, appear to be less effective than static stretches at increasing flexibility in uninjured individuals [25-29]. There is also disagreement on how long the effect of stretching lasts, although the gains in flexibility are believed to decrease relatively quickly [28,29]. Despite some evidence that those with a previous hamstring injury are significantly less flexible [30], their immediate response to stretching post warm-up has not been analysed, and a comparison with uninjured individuals has not occurred.

Traditionally, stretching is used post warm-up. de Weijer et al. [28] highlighted that no significant flexibility gains were achieved from combining warm-up and static hamstring stretches. This however, has not been examined in individuals with a previous hamstring injury and the effect on flexibility of warm-up and dynamic stretching combined is not yet known in either population group.

The aims of this study were to;

• examine the effect of warm-up, static and dynamic stretching on hamstring flexibility

• compare these effects in uninjured individuals, and in those with a previous hamstring injury

• examine if the effect on hamstring flexibility was maintained at 15 minutes post stretching.

## Methods

Ethical approval was obtained from the University of Limerick Research Ethics Committee prior to the study.

### Participants

Individuals were recruited for this study from within the university campus. All participants read an information sheet and gave written informed consent prior to participation. The previously injured subjects must have had a hamstring strain within the last year, but not within the last month. They were screened for the presence of a unilateral decrease in hamstring flexibility, to reflect the situation in clinical practice where stretching exercises are prescribed to those with reduced flexibility. A difference of at least 5 degrees in hamstring flexibility between their own injured and non-injured legs – the mean difference between injured and non-injured hamstring length in a previous study [30] – was considered sufficient for inclusion. Individuals were excluded if they were still receiving treatment, were not back to sport or full activity, or had any co-existing musculoskeletal disorders e.g. low back pain. All subjects were aged 18–40 years. Twenty individuals performed the initial assessment. Two previously injured individuals did not have a 5 degree difference in passive knee extension range of motion (PKE ROM) between limbs and were excluded. Eighteen previously injured subjects, (M = 16, F = 2), with a mean (± SD) age of 21 (± 2) years, met the criteria and completed the study. All were involved in competitive sports. 18 uninjured individuals volunteered to act as a control group. These individuals had similar characteristics to that of the previously injured group (M = 16, F = 2; Mean +/- SD age: 21 ± 1 years) and were also all involved in competitive sports.

### Study design

**Figure 1:**
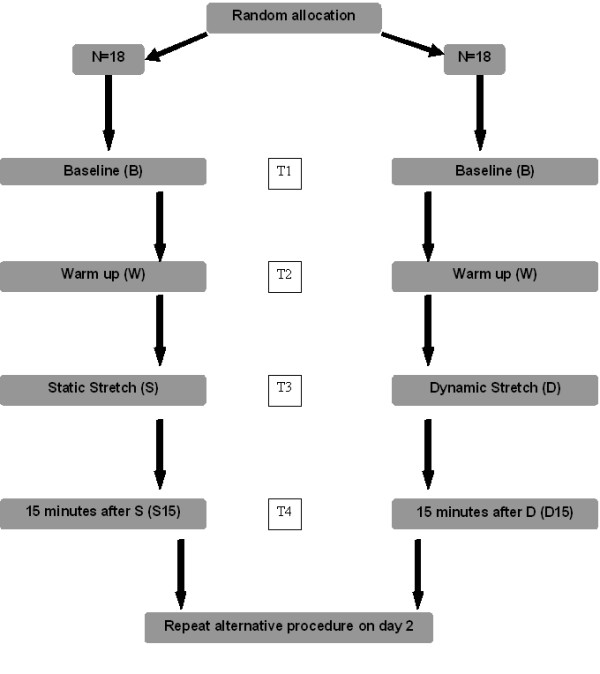
**Study design and protocol**. Subjects were randomised to order of stretching days. The same procedure was repeated on both days, apart from the stretch type (Static or Dynamic Stretching). The alternative stretch was then performed on day 2. PKE ROM was measured on 4 occasions on both days; T1- T4.

A crossover design was used. The same investigator completed all testing on both days, and all subjects underwent the same protocol apart from the order of testing. On day 1, participants randomly selected (from a sealed envelope) their group allocation for that day. Individuals followed the same protocol on both testing days, with the only difference being their allocation to either static or dynamic stretching on these days (Figure 1).

### Outcome measurement

**Figure 2:**
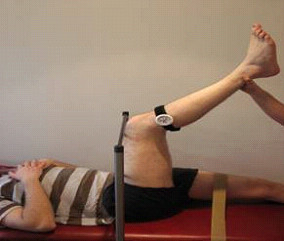
**Measurement of hamstring flexibility**. Passive Knee Extension Range of Motion (PKE ROM) was assessed with a Myrin goniometer. This procedure was repeated 3 times for each leg at all intervals and an average of all 3 measures was taken as the mean flexibility score.

Hamstring flexibility was measured using PKE ROM [31]. PKE ROM was assessed at 4 time intervals each day; (1) baseline, (2) following warm-up, (3) following stretching and (4) following a 15 minute rest period (Figure 1). Each subject was positioned in supine with the non-tested limb strapped to the plinth. The hip of the leg to be tested was passively moved to 90 degrees of hip flexion and this position was maintained through the use of a crossbar. A Myrin goniometer was placed on the lower leg in line with the fibula. The tester then extended the lower leg to its passive end of range or until the participant wanted to stop. An independent observer then recorded this measurement (Figure 2). This procedure was repeated 3 times for each leg at all time intervals and an average of all 3 measures was recorded [32].

In advance, the within-day intra-rater reliability of assessing hamstring flexibility by PKE ROM was established. Twenty-five uninjured physiotherapy students (M = 9, F = 16; mean (± SD) age = 21 (± 2.5) years), with no lower limb pathology volunteered to participate. Again, an independent observer recorded an average of 3 measurements. The intra-rater reliability was excellent (ICC = 0.945, 95%CI = -1.3078 → .6678), indicating a lack of bias and excellent test-retest repeatability [33,34]. The average standard error of measurement (SEM) based on the data was 1.84 degrees. These results indicate the reliability of the protocol was similar to or better than previous similar trials [27-29]

### Procedure

**Figure 3:**
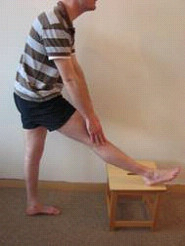
**Static stretch**. Participants placed their leg on an elevated surface with their knee extended and their ankle in plantarflexion. Each participant was then instructed to lean forward from the hip, with their spine in neutral until a stretch was felt in the posterior thigh. This position was held for 30 seconds, and repeated 3 times.

**Figure 4:**
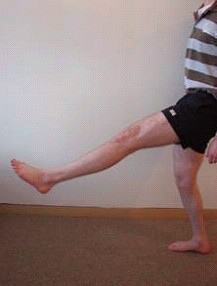
**Dynamic Stretch**. Participants actively swung the leg to be stretched forward into hip flexion until a stretch was felt in the posterior thigh whilst keeping their knee extended and their ankle in plantarflexion. This was repeated for 30 seconds, and repeated 3 times.

After randomly allocating the subject to the order of stretching days, baseline flexibility (B), as determined by PKE ROM, was measured in both legs. For the previously injured group, the injured leg was measured first. For the uninjured control group, the right leg was the first measured. Each participant then performed a 5 minute warm up [35]. Each participant jogged at a pace where 'a little breathlessness' was experienced. On completion of warm-up (W), PKE ROM was reassessed. Participants then performed their allocated stretching intervention. In the injured group, the previously injured leg was stretched first, while in the uninjured control group the right leg was stretched first. For the static stretch (S), the participant placed their leg on an elevated surface with their knee extended and their ankle plantarflexed. Participants were then instructed to lean forward from the hip, with their spine in neutral until a stretch was felt in the posterior thigh [36]. This position was held for 30 seconds, and then repeated 3 times (Figure 3). For the dynamic stretch (D), each participant was instructed to actively swing the leg to be stretched forward into hip flexion until a stretch was felt in the posterior thigh whilst keeping their knee extended and their ankle plantarflexed [35]. The leg was then allowed to swing back into slight hip extension. This was repeated for 30 seconds, such that the dynamic stretch consisted of repeated hip flexion/extension swinging movements (Figure 4). Both stretches were carried out for 30 seconds and repeated three times for each leg [28], to try to ensure that each individual carried out the same amount of stretching on both days. Once stretching was complete PKE ROM was reassessed. Participants then sat down for a 15 minute rest period prior to a final assessment of PKE ROM (S15 and D15). For each participant, testing on day 1 and day 2 was carried out at the same time of day and a period of no longer than 10 days existed between these interventions (Mean ± SD number of days between testing: Previously injured: 5 ± 2; Uninjured: 6 ± 1.75).

### Statistical Analysis

All data analysis was performed with SPSS, version 15.0. Data were normally distributed (Kolmogorov-Smirnov p > 0.05). There was no significant difference between results for the right and left legs of the control group at any time interval (p > 0.05). Therefore, an average of the 2 limbs was used to represent the uninjured control data. Further analysis was performed on 2 separate groups; previously injured legs (IN: n = 18); and uninjured control subjects (C: n = 18). The baseline (B) and post warm-up (W) PKE ROM values for each group did not differ between Day 1 and Day 2 (p > 0.05). Therefore, values from Day 1 and Day 2 for baseline and warm-up were averaged for both groups prior to analysis. One-way repeated measures ANOVA (with post-hoc Bonferroni analysis) was used to analyse differences with respect to; (1) time, (2) group and (3) time × group interactions. In addition, to reflect the slight difference between groups at baseline, ANCOVA was used to detect differences between groups.

## Results

### Interaction effect

One-way repeated measures ANOVA revealed there was no significant interaction effect (p = 0.344).

### Main effect: Time

On average across the 2 groups, there was a significant main effect for time (p < 0.001). Post-hoc testing revealed warm-up significantly increased ROM from baseline (p < 0.001), and ROM was further significantly increased after static stretching from baseline (p < 0.001) and warm-up (p = 0.04). In contrast, dynamic stretching significantly decreased ROM from warm-up (p = 0.013), although ROM remained greater than at baseline (p < 0.001). ROM after static stretching was significantly greater than after dynamic stretching (p < 0.001). After 15 minutes, there was a significant decrease in ROM for static stretching (p < 0.001), however PKE ROM was still significantly greater than at baseline for both types of stretching (p < 0.001).

### Main effect: Group

There was no main effect for group (p = 0.462), with the mean values not being significantly different at any interval (all p > 0.05). (Table 1). Using ANCOVA to adjust for the non-significant (p = 0.141) baseline differences, the previously injured group increased ROM (from baseline) more than the uninjured group after performing the warm-up and static stretching, but this difference was not statistically significant (p = 0.05).

**Table 1:** ROM values (Mean ± SD) at all intervals for both groups

Time*	**Injured (IN)**^▫^	**Control (C)**^▫^
**Baseline (B)**	142.1 ± 6.54	145.4 ± 5.84
**Warm-up (W) **‡	148.1 ± 7.04	150.1 ± 5.8
**Static (S) **‡†	151.1 ± 7.31	150.7 ± 5.34
**Dynamic (D) **‡	146.8 ± 8.59	148.1 ± 5.51
**15 mins post-static (S15) **‡	147.3 ± 7.44	148.6 ± 5.02
**15 mins post-dynamic (D15) **‡	145.6 ± 7.29	147.1 ± 5.52

^▫ ^There was no significant interaction effect, and no significant main effect for group

* There was a significant main effect for time (p < 0.001)

‡ Significant within-group increase from baseline values

† Significant within-group increase from warm-up values

## Discussion

The results of this relatively small study indicate that a gentle aerobic warm-up alone significantly increased hamstring flexibility. Static stretching also significantly increased hamstrings flexibility, whereas dynamic stretching did not. The effects of stretching reduced after 15 minutes, but flexibility remained significantly greater than at baseline. The short-term effect of warm-up and static stretching on hamstring flexibility was greater in those with reduced flexibility post-injury, but the difference was not statistically significant.

Limited literature is available to compare the combined effect of warm-up and stretching on hamstring flexibility. de Weijer et al. [28] compared the effect of warm-up and static stretching at multiple intervals up to 24 hours later on the hamstring flexibility of uninjured individuals. Results from their 4 groups (warm-up only, static stretch only, warm-up and static stretch combined, and control) showed that warm-up alone appeared to only minimally increase hamstring flexibility while static stretching alone resulted in a significant increase. The greatest increase occurred in the combined warm-up and static stretching group, but this was not significantly greater than static alone. Our results also demonstrated that combined warm-up and static stretching increased flexibility; however we did not look at static stretching in isolation and therefore cannot comment on whether this is more effective at increasing flexibility than warm-up alone. Our results for warm-up alone however, are in contrast with de Weijer et al. [28] as warm-up alone did significantly increase flexibility in the current study. Another difference is the fact that the magnitude of increase seen in our study with combined warm-up and static stretching is less than that described by de Weijer et al. [28]. There are some methodological differences between the studies, which may explain the differences. Firstly, we measured hamstring flexibility by PKE ROM, whereas they used active knee extension (AKE) ROM. Previous research has demonstrated that values obtained for hamstring flexibility using PKE and AKE vary by almost 12° [37]. This may be because AKE only measures 'initial hamstring length' whereas PKE measures 'maximal hamstring length' [37]. In addition, although the frequency and duration of static stretch was the same, the type used by de Weijer et al. [28] was slightly different.

There is considerable evidence to suggest that static stretching results in short-term increases in flexibility [26,28,29], and our results are in agreement with this. Research has also demonstrated that these increases can be maintained with regular training programs [25,27,38]. However, there has been far less research on the effects of dynamic stretching on flexibility. One previous study [39] found that static and dynamic stretching resulted in similar levels of flexibility, however the intensity of warm-up was not the same for the two stretching types, and the results are therefore difficult to compare to our study. Another study compared the effect of static and dynamic stretching on flexibility over a 6-week training programme [27]. Although Bandy et al. [27] did not look at immediate changes in flexibility, their results are similar to the current study. They also found that static stretching increased flexibility significantly more than dynamic stretching. However, their results indicated that dynamic stretching also increased flexibility, albeit not by as much (4.27° in comparison to 11.42°). This contrasts with our results, where dynamic stretching reduced post warm-up ROM. Two differences in their study, which may explain the difference in results, are the lack of a warm-up and the fact that the dynamic stretch they used contained a static element at end-range. Although the amount of research available is limited, it is difficult to justify the use of dynamic stretching as a means of increasing flexibility based on the results of both Bandy et al. [27] and the current study.

### Differences between groups

The greater increase in flexibility of the previously injured group was not statistically significant. Since this is the first study to examine hamstring flexibility in previously injured and uninjured groups, comparison with other studies is not possible. It is possible that the lack of a statistically significant difference between groups is attributable to the small sample size. In addition, the fact that the severity of injury, or the exact duration since injury, is unclear complicates interpretation of the results. Further research in groups with more clearly defined injury histories may reveal more significant differences between groups. Despite this, the increased response of the previously injured group (9° V 5.3°) may be clinically significant, and the increase in flexibility is considerably greater than the measurement error. We believe this finding is worthy of further study, since there is some limited evidence that a programme of static stretching can reduce injury risk [6,10,12,21]. In addition, static stretching may also reduce time to recovery after injury [17,18]. It is difficult to estimate the percentage of subjects with previous hamstring injury with reduced hamstring flexibility. Previous research indicates an average decrease of approximately 5° in hamstring flexibility after injury [30], and the current study excluded only two volunteers who did not display this degree of difference. Therefore, further research is needed to address some of these issues. However, it must be remembered that hamstring injury is likely to be multifactorial [40], and multiple aspects including muscle strength, endurance, agility, coordination and other factors must be also considered in it's management [21,41-44].

### Duration of effect

As stated earlier, the magnitude of increase after static stretching noted by de Weijer et al. [28] was greater than in our study, yet the pattern of decrease in flexibility after stretching was remarkably similar. They also found that the increase in flexibility reduced significantly after 15 minutes, but remained greater than baseline values, similar to our results. Of interest is the fact that the value they obtained 15 minutes after stretching remained relatively consistent when reassessed 24 hours later. In contrast, DePino et al. [29] reported that flexibility after static stretching remained significantly increased from baseline for only 3 minutes post stretching. This is in contrast with the current study, where after 15 minutes flexibility was still greater than baseline. Differences in how the pre-stretch baseline measurements were recorded may explain this. DePino et al. [29] allowed subjects to perform 5 AKE's prior to a 6^th ^being taken as the baseline measurement, as a form of warm-up to eliminate variability in measurements. In contrast, in our study we took the baseline measurement as an average of the first 3 measurements, to ensure an accurate 'cold' baseline. This may explain why their hamstring flexibility values at 15 minutes were actually below their 'baseline' measurements (1.1° for intervention, 6.5° for control) [29]. This would suggest that their 'baseline' measures were similar to our warm-up values, for which the results of the studies are similar. Results from other studies also indicate that the effect of stretching lasts somewhere between 6 and 25 minutes [45,46]. It appears therefore that the effect of both types of stretching reduces quickly, but remains higher than baseline.

### Impact on performance

It is important to acknowledge that flexibility is not the only parameter of interest which may be influenced by stretching. There is consistent evidence that dynamic stretching improves performance measures such as agility, speed and strength whereas static stretching may actually decrease performance [2,4,24,47-50]. Therefore, the choice of stretching may depend on the aims of rehabilitation e.g. to increase flexibility or other parameters of interest e.g. power. It appears that flexibility improves most with static stretching, whereas immediate physical performance improves most with dynamic stretching. Ross [51] suggested that a 'purposeful delay' after static stretching appears to alleviate the negative effects on performance, and recommended that static stretching used in this manner can actually improve performance long-term [2,45,51,52]. Booth [1] suggested that warm-up and stretching immediately pre-participation should focus on performance aspects, rather than improving flexibility, and therefore static stretching should be done at times other than pre-participation.

### Limitations

The study design did not allow us to determine whether warm-up or static stretching had the greatest effect on flexibility. Both were performed on the same day to reflect the pre-participation situation, however further studies may differentiate between the effect of these factors. The warm-up only used a subjective instruction to the subjects to guide intensity, which limits reproducibility. The exclusion criteria meant that not all subjects with previous hamstring strain were eligible for the study. Therefore the results cannot be extrapolated to all athletes with previous injury e.g. those with good flexibility post-injury. Similarly, the participants were mostly young and male, and further study is needed in more diverse populations. The small sample size limits the conclusions that can be made, and further larger studies are needed. The sample size is however similar to previous similar trials [29,39] and the number in each group is in line with previous studies [28]. All subjects were seated for 15 minutes on a standard chair with their feet on the floor after stretching, however their exact knee and hip angles were not standardised, which could have influenced the results after 15 minutes rest. Neither the subjects nor investigator were blinded to the stretching interventions performed. This bias was minimised however by the use of an independent observer to measure the ROM. We acknowledge that other research indicates these short-term increases are only maintained by following a suitable ongoing training programme [53]. We attempted to have both groups perform a similar magnitude of stretching (3 × 30 seconds), however the nature of dynamic stretching means the dynamic group spent less time in a lengthened position. In addition, the number of repetitions of the dynamic stretch performed may have varied between individuals. Despite this, the amount of dynamic stretching was standardised to time to allow comparison with the static stretch, and to reflect the reality of using dynamic stretching in clinical practice. The alternative of asking each participant to perform a defined number of leg swings could have caused considerable variation in the duration of stretching performed, due to individual variations in the rate of leg swings performed. As stated already, this study considered only the effect stretching had on hamstring flexibility. It is acknowledged that other factors apart from flexibility (e.g. immediate performance) must be taken into consideration when choosing the type of stretching programme suitable for a client. No objective measure was available to verify the history of hamstring strain e.g. ultrasound. However, self-report has shown to be moderately valid for diagnosis of previous injury [54] and been used in previous research on hamstring injury [55,56]. In addition, the length of time since injury, the duration of time away from sport, or rehabilitation protocols used since injury are unknown. Subjects with an injury in the past month were excluded as it was felt that natural recovery of ROM after injury in these subjects may have confounded the results. Similarly, those with no injury in the past 12 months were deemed to be less likely to display reduced flexibility. We did not examine all static and dynamic stretching techniques, and results may vary using different stretching protocols. There is still considerable debate about the number of repetitions and duration of stretches that is optimal [1,36,57], but the protocol chosen here reflects common clinical practice. This study did not examine the effectiveness of other methods of increasing hamstring flexibility. Further studies are needed to investigate the role of other means of increasing hamstring flexibility e.g. eccentric training [58] or other types of stretching e.g. PNF [59]. This is particularly significant as eccentric training can also influence other potential risk factors (e.g. muscle strength) during rehabilitation [60]. This study did not examine if increased flexibility resulted in an improved clinical outcome. Despite these limitations, the strengths of the study must be acknowledged. It would appear that the current study is the first to assess the effects of warm-up and static/dynamic stretching on hamstring flexibility in both previously injured individuals and closely matched uninjured controls. The reliability of the protocol was established, and the inclusion/exclusion criteria were clear and clinically relevant.

## Conclusion

An active aerobic warm-up significantly increased hamstring flexibility. After warm-up, static stretching further increased flexibility while dynamic stretching decreased flexibility. Gains in flexibility reduced after 15 minutes rest, but flexibility remained significantly greater than at baseline. The previously injured subjects demonstrated a greater increase in flexibility after warm-up and static stretching than the uninjured control subjects, however this did not reach statistical significance. The results indicate that static stretching should be performed if the aim is to increase flexibility, in line with previous research. Further research is needed to determine the significance of reduced flexibility, the role of stretching in reducing injury risk, and the mechanism of action of stretching in increasing ROM [61-63].

## Competing interests

The authors declare that they have no competing interests.

## Authors' contributions

KOS was involved in conception and design of the study, data analysis and interpretation, as well as drafting and editing the final document for publication. EM was involved in conception and design of the study, data collection, data analysis and interpretation, as well as drafting and editing the final document for publication. DS was involved in conception and design of the study, data interpretation, as well as drafting and editing the final document for publication. All authors read and approved the final manuscript.

## Pre-publication history

The pre-publication history for this paper can be accessed here:


